# A Regional Tele-Paediatric Network (RTP-Net) in the northeast of Germany—results of an implementation study

**DOI:** 10.1186/s12913-025-13097-7

**Published:** 2025-09-17

**Authors:** Luisa Tischler, Nils Pfeuffer, Yvonne Jordan, Sarah Heimbuch, Heiko Krause, Angelika Beyer, Maria Zach, Astrid Bertsche, Udo Gesser, Markus Krohn, Steffen Fleßa, Wolfgang Hoffmann, Neeltje van den Berg

**Affiliations:** 1 Section Epidemiology of Health Care and Community Health, Institute for Community Medicine, University Medicine Greifswald, Ellernholzstr. 1-2, 17489 Greifswald, Germany; 2 German Center for Child and Adolescent Health (DZKJ), Partner Site Greifswald/Rostock, Greifswald, Germany; 3 Institute for Nursing Science and Interprofessional Learning, University Medicine Greifswald, Greifswald, Germany; 4 District Hospital Wolgast, Wolgast, Germany; 5 Department of Neuropaediatrics, Clinic and Polyclinic for Child and Adolescent Medicine, University Medicine Greifswald, Greifswald, Germany; 6 Paediatric Clinic Department, Sana Hospital Rügen, Bergen, Germany; 7 Chair of General Business Administration and Health Care Management, University of Greifswald, Greifswald, Germany

**Keywords:** Paediatrics, Telemedicine, Regional care, Network, Healthcare services research

## Abstract

**Background:**

Mutual support of paediatric hospital departments through a telemedical network can support paediatric care particularly in rural areas with structural deficits. Using a participatory approach, a *Regional Tele-Paediatric Network* (RTP-Net) was developed and implemented in the northeast of Germany. The tele-paediatric network provides the following functionalities: 1) telemedical triage, 2) subspecialist (video) consultation, 3) virtual background services (24/7) and 4) video consultation with patients at home and was available both for patients of the emergency room and for inpatients. The aim of the present analysis was to identify which functionalities of the tele-paediatric network were most frequently used and in which diagnostic and treatment contexts they were applied. Additionally, the study examined the impact of telemedicine consultations for paediatric patients on their subsequent treatment.

**Methods:**

Patients under 18 years of age who received telemedical treatment in a participating hospital during the observation period from February 2021 to March 2024 were included in the analysis. The telemedical contact was documented by both, the clinic on site and the telemedicine contact clinic, in a digital patient file on a shared documentation platform (eHealth platform). The documentation included patient data and documents and the diagnostic and treatment recommendations resulting from each telemedical contact quantitative data on utilization, reasons for the telemedicine consultation, and treatment consequences of the telemedical contact were collected using electronic case report forms and were then analysed descriptively.

**Results:**

In the study period, 146 physicians from 13 hospitals participated in the development and implementation of RTP-Net (ranging from small hospitals for basic care to university hospitals). *N* = 403 paediatric patients (thereof 198 female patients) with a total of 507 cases were included, resulting in 519 telemedical contacts. Telemedicine was particularly used for children under 3 years of age (*n* = 150/403; 37.2%). The most frequently used functionality was the subspecialist consultation (*n* = 290/519; 55.9%), followed by virtual background duty services (*n* = 169/519; 32.6%), which were often used as a supplement to the internal on-call duty service. The most common reason for tele-paediatric care were consultations to evaluate symptoms and findings in children with diseases of the nervous system (ICD-10-GM: G00-G99) (*n* = 161/507; 31.8%), e.g. the joint evaluation of Electroencephalographies (EEGs). In 51/519 cases (9.8%), a hospital admission at the local clinic followed the telemedicine consultation, and in 27/519 cases (5.2%), the patient was transferred to the telemedicine contact clinic. The most frequent outcome was a change in the ongoing treatment (*n* = 198/519; 38.2%). Hospital admission (*n* = 51/519) was more common for younger children (median age 6 years) with respiratory system diseases (*n* = 13), whereas for older children (median age 8 years) with nervous system diseases (*n* = 124) the telemedicine physician mostly recommended therapy adjustments and these patients usually remained at the clinic on site (*n* = 198).

**Conclusion:**

The *Regional Tele-Paediatric Network* is suitable to support regional paediatric healthcare. Neuropaediatric cases are frequent but often have nonspecific symptoms, which would otherwise require transfer to a more specialized clinic. In the network most paediatric patients could be treated at the clinic on site (local clinic) without needing to seek care at a distant hospital. This avoids long travel distances for the patients and provides economic benefits to the clinic on site, which can continue the treatment of the child locally.

**Trial registration:**

This study was registered under the title ‘Implementation und Evaluation of a Regional Tele-Paediatric Network in Mecklenburg-Western Pomerania and Brandenburg’ (registration no. DRKS00024002, https://drks.de/search/de/trial/DRKS00024002) at the German Registry for Clinical Trials (date of registration 07.01.2021).

## Introduction

### Background

In rural areas of Germany with low population density, maintaining inpatient paediatric care and emergency services is increasingly challenging. The German Diagnosis-Related (G-DRG) system inadequately reimburses many paediatric services, putting inpatient care under financial pressure. In rural regions, low case numbers often lead to centralisation or service gaps [1]. Additionally, demographic ageing has led to a growing shortage of healthcare professionals, limiting service provision and access. As a result, the number of paediatric practices is declining, and many paediatric departments and children’s hospitals have closed due to economic or staffing issues [2, 3]. Consequently, hospitals without paediatrics no longer provide paediatric emergency care. These developments underscore the need to maintain effective paediatric emergency services, especially in rural hospitals.

Against the backdrop of these structural deficits, the development of innovative, regional, and integrative care models to ensure comprehensive paediatric care has become increasingly urgent. The ongoing digitalization of healthcare in recent years has enabled and accelerated the implementation of telemedicine technologies in various areas of medical care. Particularly in paediatric care, telemedicine, usually defined as the provision of medical services via telecommunications [4], plays an increasingly important role. Children and adolescents often require close and specialized care due to their specific needs and conditions - a challenge that is further amplified by the scarcity of certain specialisations in rural regions, where telemedical applications can help ensure more timely and flexible care regardless of geographical barriers. In inpatient paediatric care, telemedicine offers promising approaches to improve the quality of care, efficiency, and patient safety [5]. Telemedicine functionalities encompass a variety of digital applications, including video consultations, telemetric monitoring, and AI-based decision support, which can facilitate access to specialized physicians and overcome spatial and temporal barriers [6].

International studies demonstrate the added value of telemedicine approaches in paediatrics. Research has shown that telemedicine consultations in neonatal intensive care as a means to follow up infants after discharge can decrease the need of hospital visits [7] and prevents unnecessary transfers to a higher level of care [8]. In a systematic review Mitra et al. revealed that telemedicine enhances paediatric emergency care provision, enhances therapeutic decision-making, improves diagnostic accuracy, and reduces costs [9]. In paediatric acute care, telemedical triage in a rural region in the northeast of Germany has proven to be a promising option for supporting paediatric care [1, 10]. Studies on telemedicine for children and adolescents with chronic illnesses demonstrate positive effects on the management of care for children with special needs [11]. Patient and family satisfaction with telemedicine services is high [12–16].

**Fig. 1:**
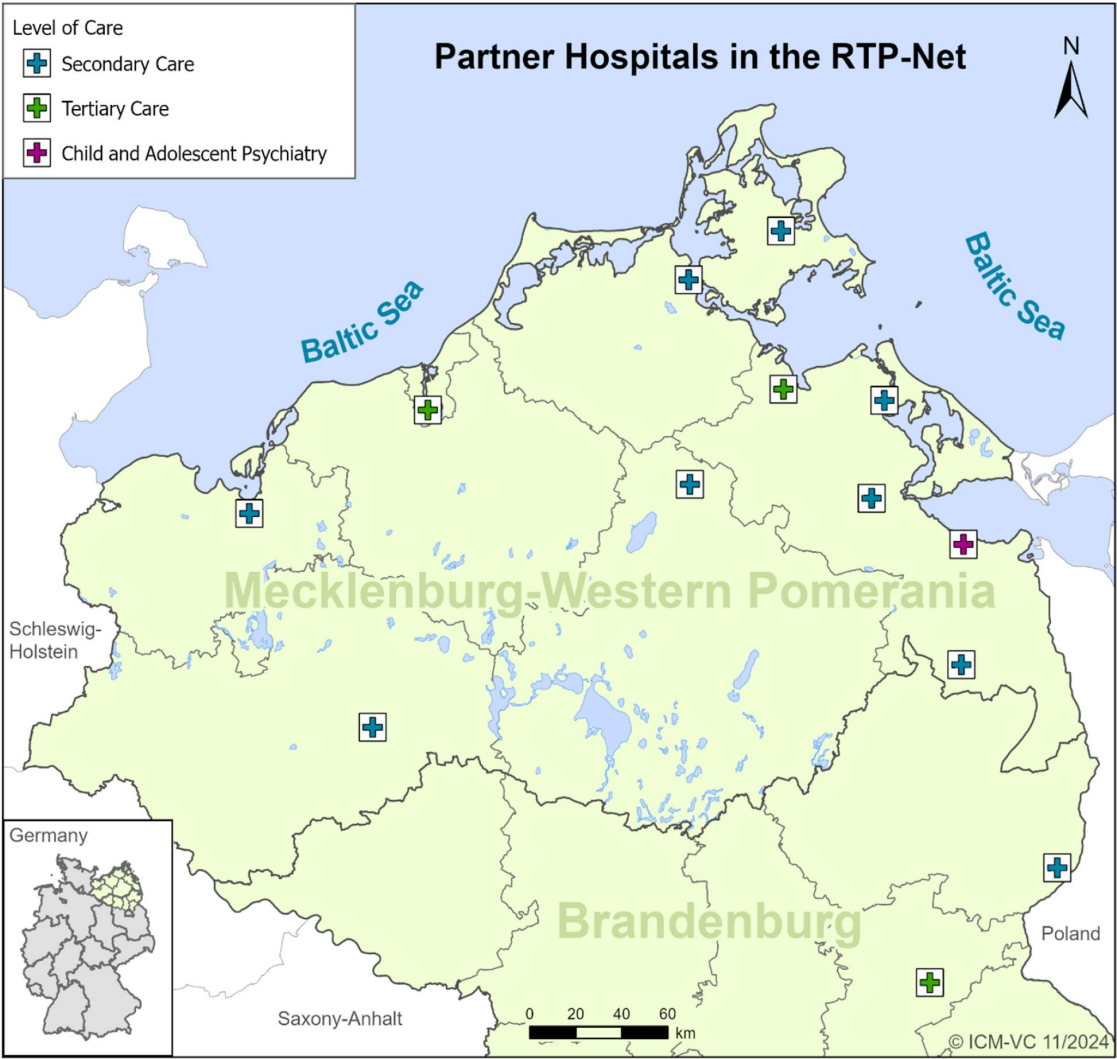
Participating partner hospitals in the RTP-Net (Secondary Care: Specialized paediatric medical services provided in hospitals or clinics, Tertiary Care: Highly specialized paediatric medical treatment offered in major hospitals or university medicals centers, Quaternary Care: The highest level of specialized care, available in selected research hospitals)

While telemedicine is increasingly being integrated into paediatric healthcare, most existing studies have focused on individual applications and were often conducted in controlled research settings. In Germany, there are only a few telemedicine networks in adult medicine that are sustainably integrated into care [17, 18]. In paediatrics, the Paediatric Intensive Care Network in Lower Saxony (PIN) is currently in the process of gradually implementing telemedical applications [19]. Additional paediatric telemedicine networks are still in the research or project phase [20–22]. There remains a lack of comprehensive data on how telemedical functionalities are actually used in routine paediatric clinical practice - particularly across different paediatric subspecialties and diagnostic contexts in inpatient settings. Moreover, utilisation patterns and clinical integration in structurally underserved, rural regions have so far been insufficiently studied.The Regional Tele-Paediatric Network (RTP-Net) was developed to address the increasingly strained situation in inpatient paediatric care in the northeast of Germany (Federal States of Mecklenburg-Western Pomerania and Brandenburg). The network was implemented in an inpatient paediatric care setting and includes 13 hospitals with paediatric departments of different sizes and providing different levels of care and specialties (see Fig. 1). The tele-paediatric network provides 4 functionalities: 1) telemedical triage, 2) subspecialist (video) consultation 3) virtual background services for the participating hospitals and 4) video consultation with patients at home.

## Objective

The aim of the RTP-Net project was to develop and implement a regional telemedicine network to support and ensure inpatient paediatric care in a rural region in the northeast of Germany. The following publication pursues the main research question: Which telemedical functionalities of the tele-paediatric network are used, how often and in which diagnostic and treatment situations?

- What effects do telemedical contacts have on the further treatment of paediatric patients?

## Methodology

### Study design

The implementation of the tele-paediatric network was conducted as a prospective, non-controlled study under real-world conditions. A key aspect was the participatory research approach (CBPR) [23], which promotes the adaptation of the healthcare concept, the implementation and the enhancement of documentation through close collaboration with the affected stakeholders, leading to practical insights and real-world evidence. This approach enables an individualized consideration of the needs of users and the development of tailored solutions. As a result, CBPR enhances the relevance and applicability of the research findings in real-world contexts. In this context, parental perspectives were also incorporated via a complementary mixed-methods study assessing satisfaction and acceptance of telemedicine, providing valuable end-user feedback to inform the evaluation and future development of the intervention [16].

In RTP-Net, the telemedicine functionalities described in Table 1 were developed and made available to the users.

**Table 1:** Telemedical functionalities in RTP-Net

Functionality	Definition
Telemedical Triage	Standardized assessment of the treatment urgency for children who visit a hospital emergency department without an available paediatrician
Subspecialist (video) consultation	Consultation between two physicians regarding specialized medical questions, e.g. the evaluation of medical images of diagnostic outreads, when the subspecialist is not available on-site
Virtual background service	Assumption of the specialist support role and teleconsultation by a paediatrician. During the project period, this function did not fully replace the specialist on-call duty service
Video consultation with patients at home	Consultation between physician and their patient at home, e.g. to discuss medical findings

Physicians involved in the telemedicine healthcare concept exchanged patient health information (both structured data and *.pdf-files) via a web-based regional patient record, the so-called eHealth platform of the University Medicine Greifswald. The eHealth platform consisted of an Orchestra eHealth Suite database server at the backend (Orchestra eHealth Suite; Version 18.2.1; x-tention, Wels, Austria) and the C37-CaseBoard at the frontend (celsius37.com AG, Mannheim, Germany). After each tele-medicine consultation, the participating physicians in RTP-Net were asked to complete two electronic contact forms within the eHealth platform. One contact form was used to document the presentation date, hospital location, patient history, reason for consultation, and the duration of the telemedical interaction. The second contact form included questions to assess user satisfaction with telemedicine and any technical and organizational issues that arose during the consultations. Video consultations were conducted through the video consultation provider arztkonsultation.de (https://arztkonsultation.de, arztkonsultation ak GmbH, version 2.26.8, Schwerin, Germany).

The project began April 1, 2020, and ended March 31, 2024. The observation period (recruitment period) following the technical implementation of the telemedicine functionalities and the training of participants lasted 35 months (April 2021 to February 2024).

### Participants

#### Hospitals

All interested hospitals with paediatric departments in Mecklenburg-Western Pomerania and northern Brandenburg were eligible to participate. Legal basis for participation was a signed cooperation agreement. This agreement allowed physicians working in the paediatric departments to list their medical profile and contact information on the project’s website to be available for telemedicine inquiries and consultations. The participating hospitals were responsible for recruiting patients for the implementation study. Each hospital could serve as both a requesting clinic for telemedicine consultations and as a consulting hospital. In the following text, the “clinic on site” refers to the participating hospital where the respective patient presented and was recruited for the study. The “telemedicine contact clinic” refers to the participating clinic where a physician was asked for a telemedicine consultation.

#### Healthcare providers/physicians

Healthcare providers were physicians who were employed at a participating clinic during the project period and who received training and access credentials (eHealth platform, video consultation provider, project homepage).

#### Patients

The study included patients under 18 years of age who presented to participating paediatric departments or emergency rooms. Parents were informed about the study and asked to provide written informed consent for any potential telemedicine intervention and the use of data for research purposes. Participation was voluntary and integrated into routine clinical care. Eligible patients could be included based on clinical need, particularly when additional paediatric expertise was required. Importantly, inclusion in the study was not a prerequisite for receiving care through the tele-paediatric network. All patients were treated regardless of study participation. Treating physicians could exclude patients for medical reasons, such as high urgency or the need for immediate resuscitation. Patients aged 18 years or older were excluded from the analysis. The proportion of paediatric patients who presented at participating hospitals but were not enrolled in the study remains unknown.

### Outcomes

#### Use of functionalities

For this outcome, the item “Which type of telemedicine functionality/consultation did you use for this patient?” was derived from the contact form of both the clinic on site and the telemedicine contact clinic for each case. The choices included: 1) Telemedical triage, 2) Virtual background service, 3) Subspecialist (video) consultation, and 4) Video consultation with patients at home.

#### Diagnoses prior to the telemedicine contact

Here, the suspected diagnosis (ICD-10-GM 2024, German Diagnosis related Groups) [24] of the paediatric patient was derived from the contact form of the clinic on site. Additionally, the (preliminary) treatment diagnosis for each case was derived from the contact forms of the telemedicine contact clinic and compared with the preliminary diagnosis prior to the telemedicine consultation.

#### Consequences of the telemedicine contacts

From the contact forms of the telemedicine contact clinics, the item "Consequences of the consultation" was abstracted for each case. The available options were: a) Referral to a paediatric emergency service of the *Association of Statutory Health Insurance Physicians*, Paediatric Portal Practice Clinic, or similar (outside of regular practice hours), b) referral to a general practitioner or a paediatrician´s practice during regular practice hours, c) hospital admission, d) transfer to another hospital, e) patient can be treated in the clinic on site but change in ongoing treatment, f) no action.

### Data assessment and analysis

Data were collected using electronic case report forms (eCRFs) on the eHealth platform and extracted from the platform after the observation period. Data were evaluated descriptively. Data analysis and visualization were carried out using Microsoft Excel (Microsoft Office LTSC Professional Plus 2021) and the statistic program package SAS (Version, Fa, Ort JMP). The data analysis followed the"Good Epidemiological Practice"guidelines [25].

## Results

**Fig. 2:**
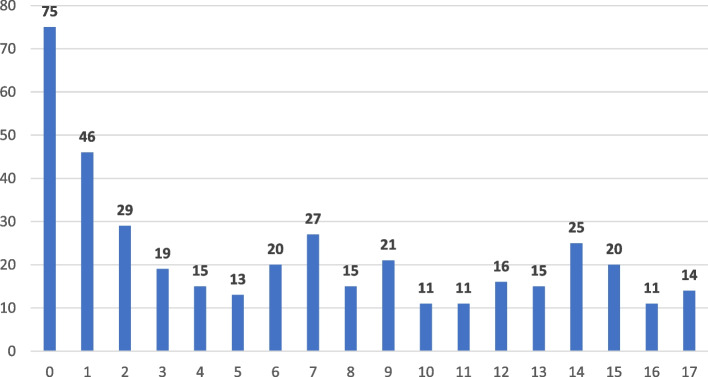
Distribution of the age of the patients (0–17 years) in the RTP-Net (*N* = 403)

The tele-paediatric network started with 6 hospitals, and throughout the course of the project, additional hospitals joined, increasing the total number of hospitals integrated into the network to 13. Shortly after the start of the project, at the request of some physicians, the functionality"video consultation with patients at home"was added. During the project period, a total of 146 physicians participated in the project, i.e. were provided with access to the eHealth platform, the video consultation provider, and the project homepage after training. The network offered 17 paediatric subspecialisations (paediatric hematology and oncology, paediatric cardiology, neonatology, paediatric neurology, paediatric endocrinology, paediatric diabetology, paediatric gastroenterology, paediatric nephrology, paediatric pneumology, paediatric rheumatology, paediatric orthopedics, diagnostics/treatment for insect venom allergy, paediatric radiology, allergology, paediatric surgery, treatment of vascular malformations, myalgic encephalomyelitis). Between April 2021 and February 2024, the participating physicians recruited 431 patients. *N* = 4 patients were excluded since they were older than 18 years and *n* = 24 patients were excluded since no telemedical contact took place. Of the 403 patients (507 cases) included, 198 (49.1%) were female. The median age of the patients was 6.17 years (IQR: 1.42–12.00), with a range from 0 to 17 years (mean ± SD: 6.82 ± 5.67 years) (see Fig. 2). Telemedicine was mostly used to supplement treatment for younger children under 3 years of age (*n* = 150; 37.2%).

### Utilization of telemedical functionalities

**Fig. 3:**
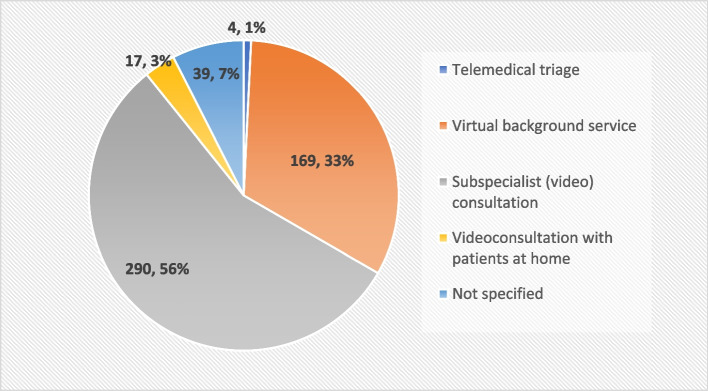
Use of telemedicine functionalities (*N* = 519 telemedical contacts)

The total number of documented cases differs between the clinic on site (*N* = 507) and the hospital providing the telemedical contacts (*N* = 519), since several telemedical contacts were conducted for some of the patients, sometimes on the same day. Figure 3 shows that the most frequently used functionality of the paediatric telemedicine network was the telemedical subspecialist consultation (*n* = 290/519; 55.9%), followed by the virtual background service (*n* = 169/519; 32.6%). The video consultation with patients at home was added later in the project and was primarily utilized by one physician (*n* = 17/519; 3.3%). Telemedical triage was rarely applied within the project (*n* = 4/519; 0.8%). By far, the most frequently requested subspecialisation for specialist consultations was paediatric neurology (*n* = 265/519; 50.0%), followed by neonatology (*n* = 19/519;3,7%) and paediatric surgery (*n* = 19/519; 3,7%. In 74 cases, the patient was present during the telemedical contact (*n* = 74/519, 14.3%), particularly when the telemedical functionality of a virtual background service was used (*n* = 56/74; 77.0%).

### Suspected diagnoses prior to the telemedicine contact

The clinics on site documented a total of 134 different ICD-10 codes. Table 2 shows that paediatric patients were primarily treated in the clinics on site for diseases of the nervous system (ICD-10 G00-G99; n = 161/507; 31.8%) and for symptoms and abnormal clinical laboratory findings not classified elsewhere (ICD-10 R00-R99; *n* = 49/507; 9.7%). For 178 (35.1%) cases, no suspected diagnosis was documented. Among the patients treated for a nervous system disorder (ICD-10 G00-G99), the clinics on site made the suspected diagnosis "epilepsy" in 86.3% of the cases (*n* = 139/161) and "migraine" in 7.5% (*n* = 12/161). Among the patients with the diagnosis"symptoms and abnormal clinical laboratory findings, not classified elsewhere"(ICD-10 R00-R99), 44.9% (*n* = 22/49) were suspected to have"seizures, not classified elsewhere."In 12.2% of patients (*n* = 6/49), the suspected diagnoses of"headaches"were documented, and in 10.2%"syncope and collapse"(*n* = 5/49).

**Table 2:** ICD-10: Suspected Diagnosis Prior to Telemedical Contact (*N* = 507)

Suspected Diagnosis (ICD-10)	Frequency (n; %)	Median age of the patients (years)	Range age (years)	Female (n; %)
Certain Infectious and Parasitic Diseases (A00-B99)	15 (3.0)	1	0–9	7 (46.7)
Neoplasms (C00-D48)	2 (0.4)	0.5	0–1	0 (0.0)
Diseases of the Blood and Blood-Forming Organs and Certain Disorders Involving the Immune Mechanism (D50-D90)	1 (0.2)	6	/	1 (100.0)
Endocrine, Nutritional, and Metabolic Diseases E00-E99)	3 (0.6)	0	0–1	1 (33.3)
Mental and Behavioural Disorders (F00-F99)	8 (1.6)	9.5	5–17	4 (50.0)
Diseases of the Nervous System (G00-G99)	161 (31.8)	8	0–16	84 (52.2)
Diseases of the Circulatory System (I00-I99)	7 (1.4)	4	0–17	2 (28.6)
Diseases of the Respiratory System (J00-J99)	28 (5.5)	1	0–16	10 (35.7)
Diseases of the Digestive System (K00-K93)	7 (1.4)	10	7–17	6 (85.7)
Diseases of the Skin and Subcutaneous Tissue (L00-L99)	2 (0.4)	5.5	2–9	0 (0.0)
Diseases of the Musculoskeletal System and Connective Tissue (M00-M99)	1 (0.2)	4	/	1 (100.0)
Diseases of the Genitourinary System (N00-N99)	6 (1.2)	2.5	0–3	5 (83.3)
Certain Conditions Originating in the Perinatal Period (P00-P96)	6 (1.2)	0	0	4 (66.7)
Congenital Malformations, Deformations, and Chromosomal Abnormalities (Q00-Q99)	10 (2.0)	0	0	6 (60.0)
Symptoms and Abnormal Clinical and Laboratory Findings Not Classified Elsewhere (R00-R99)	49 (9.7)	7	0–17	22 (44.9)
Injuries, Poisonings, and Certain Other Consequences of External Causes (S00-T98)	18 (3.6)	9	0–15	8 (44.4)
Codes for Special Purposes (U00-U99)	1 (0.2)	0	0	1 (100)
Factors Influencing Health Status and Contact with Health Services (Z00-Z99)	4 (0.8)	0	0	1 (25)
No information (data)	178 (35,1)	6	0–17	94 (52.8)
Total	507 (100.0)	7	0–17	257 (50.7)

In 298 out of 519 cases, a diagnosis was documented both before and after the telemedicine contact in the eHealth platform. The suspected diagnoses made by the local clinic prior to the telemedicine consultation and the (preliminary) treatment diagnoses established by the telemedicine consultation clinic after the consultation differed in 57.7% of cases (n = 172/298), as diagnoses were often adjusted or refined during the telemedicine contact. 126/298 diagnoses (42.3%) were identical. in 73/172 (42.4%) adjustments the complete diagnostic group was changed. This most frequently concerned changes between diagnosis groups G and R (*n* = 33). In 221/519 cases (42.6%), no comparison was possible due to missing documentation in the eHealth platform.

### Consequences of the telemedicine contact

The telemedicine contact clinics most common category reported that the ongoing treatment was modified as a consequence of the telemedicine consultation (*n* = 198/519; 38.2%). In 16.6% of the cases,"other"(*n* = 86/519) were indicated."Other"included, among other things, the recommendation of further diagnostics (e.g., EEG, cMRI), follow-up consultations within a specified time, or recommendations for a wait-and-see approach. In 10.2% of the cases (*n* = 53/519), no other or further treatment steps were necessary ("nothing") and in 9.8% of the cases, a hospital admission occurred (*n* = 51/519). In few cases, the patient was referred to the general practitioner or a private paediatrician during regular office hours (*n* = 39/519; 7.5%) or transferred to another hospital (*n* = 27/519; 5.2%). In 12.3% of the cases (*n* = 64/519), no information was provided (see Table 3).

**Table 3:** Consequences of telemedicine

Consequences of telemedicine (n; %)	Frequency n (%)	Median age (years)	Female n (%)	Most common diagnosis (TOP 3)
Modification of ongoing treatment	198 (38.2)	8	107 (48.6)	diseases of the nervous system (*n* = 124); symptoms and abnormal clinical and laboratory findings not classified elsewhere (*n* = 42); diseases of the respiratory system (n = 4)
Other	86 (16.6)	6	47 (54.7)	diseases of the nervous system (*n* = 30); symptoms and abnormal clinical and laboratory findings not classified elsewhere (*n* = 11); injuries, poisonings, and certain other consequences of external causes (*n* = 9)
No information provided	65 (12.3)	5	31 (47.7)	diseases of the respiratory system (*n* = 8); certain infectious and parasitic diseases (n = 5); diseases of the nervous system (*n* = 5)
No further treatment steps	53 (10.2)	2	23 (43.4)	diseases of the respiratory system (*n* = 10); Injuries, poisonings, and certain other consequences of external causes (*n* = 9); diseases of the nervous system (n = 7)
Hospital admission	51 (9.8)	6	28 (54.9)	diseases of the respiratory system (*n* = 13); symptoms and abnormal clinical and laboratory findings not classified elsewhere (*n* = 11); diseases of the nervous system (n = 6)
Referral to general practitioner or paediatrician in private practice during regular office hours	39 (7.5)	2	20 (51.3)	diseases of the nervous system (*n* = 9); certain infectious and parasitic diseases (n = 8); injuries, poisonings, and certain other consequences of external causes (n = 6)
Transfer to another hospital	27 (5.2)	6	8 (29.6)	diseases of the nervous system (*n* = 13); mental and behavioural disorders (n = 2); congenital malformations, deformations, and chromosomal abnormalities (n = 2); symptoms and abnormal clinical and laboratory findings not classified elsewhere (n = 2); injuries, poisonings, and certain other consequences of external causes (n = 2)
Referral to paediatric emergency service of the health insurance association, Paediatric Portal Practice Clinic, or similar (1; 0.2)	1 (0.2)	1	0 (0.0)	injuries, poisonings, and certain other consequences of external causes (n = 1)

Table 3 shows that referrals to the outpatient sector during regular office hours (n = 39; median age: 2 years) or cases in which no further treatment steps were necessary after the telemedicine consultation (*n* = 53; median age: 2 years) were more common in younger children. Treatment adjustments (modifications of ongoing treatment) were more frequently made for older paediatric patients (n = 198; median age: 8 years), often for diseases of the nervous system (*n* = 124).

## Discussion

In the present analysis, we aimed to determine which telemedical functionalities were most frequently used within a regional tele-paediatric network and what consequences telemedical contacts had for further treatment. Our results indicate that specialist video consultations emerged as one of the most frequently used telemedicine applications throughout the project. Particularly in the field of paediatric neurology, such as for the evaluation of EEGs and the corresponding initiation of therapies, this service was well received by both the involved physicians and patients. These cases are common in the clinic and often present with nonspecific symptoms that would otherwise require transfer to another clinic. A striking finding is the high proportion of neurological diagnoses—particularly epilepsy—among the telemedicine cases. This is partly explained by the frequent use of subspecialist video consultations in paediatric neurology, especially by one participating hospital that serves as a certified epilepsy centre. Sotoodeh et al. provide an overview of the successful application of EEG in telemedicine and highlight the discrepancy between the frequent need for EEG recordings and the lack of comprehensive personnel resources, which creates a growing demand for telemedical interpretation [26]. This reflects the specific clinical utility of telemedicine in neuropaediatrics, where timely EEG interpretation and specialist input are essential. However, this concentration of cases may limit the generalisability of the findings, as the diagnostic spectrum is not evenly distributed across all participating sites.

This finding demonstrates that in highly specialized fields, where expert knowledge is scarce and not available at every location, telemedicine is an effective complement to traditional outpatient consultations by enabling access to such expertise and supporting precise clinical assessments remotely. In many of the cases, the use of telemedicine made it much easier to provide prompt and well-founded advice, which had a positive effect on the care of children with neurological disorders, e.g., by avoiding transferrals and thus enabling a continuity of care for children locally close to their home.

Particularly physicians who dealt with rare or chronic diseases in children and adolescents expressed a desire for an addition to video consultations with their patients at home, especially for preliminary discussions or consultations on the results. Initial studies have already highlighted the benefits such as time and cost savings and improved the quality of medical care [27, 28]. This desire for a stronger integration of telemedicine solutions in the management of complex and long-term disease trajectories underscores the value of telemedicine in the care of children with special needs, although further structural development appears necessary to better meet physicians' expectations.

The "virtual background" functionality was rarely used between different hospitals throughout the project. Instead, this functionality was more frequently utilized within individual hospitals, for example, for consultations between junior doctors and senior consultants on call or for handovers during shift changes or after weekend duties. The limited inter-hospital use of this option was mainly due to the decentralized organization of the hospitals and the associated difficulties in reliably reaching the responsible individuals. Additionally, some reluctance was observed, as not involved parties were well acquainted with each other. This lack of familiarity hindered effective collaboration across hospital boundaries and led to uncertainties in strengths and limitations using the virtual background as a communication tool. Furthermore, differences in internal processes and procedures across hospitals made coordinated and standardized use of telemedicine more difficult. A central organization, along with a more unified structure, and clear, standardized procedures, could potentially alleviate these issues in the future, lowering the barriers to inter-hospital usage and fostering more effective collaboration.

Telemedical triage was used only very rarely within the framework of the project, which may indicate either a currently low demand or potential barriers to its practical implementation. The integration of this telemedicine functionality revealed that it was perceived by users as impractical and not necessarily essential. This may have been due to the fact that all participating hospitals had a paediatric department. Furthermore, according to some users, the distinction between telemedical triage and virtual background services was not entirely clear. Additionally, although the nurses responsible for triage were trained, they were less consistently involved in the project due to a lack of resources (time, interest). However, Beyer et al. demonstrated in a previous project in the same region that telemedicine-based urgency assessment in paediatric acute care is a promising option to support care delivery in a hospital, where the paediatric ward had been closed [1]. This reveals the importance of context for the practical applicability of telemedicine services. Particularly in acute medicine, where quick and clear decisions need to be made, many physicians consider the personal examination to be irreplaceable [29, 30]. The reluctance to use telemedical triage may indicate that telemedicine, especially in the field of acute care, is not yet recognized as an adequate alternative.

Over the course of the project, it became evident that the telemedical consultations in many cases allowed a specification of the initial suspected diagnosis as well as a modification of the ongoing treatment. Many of the reasons for changes in treatment were of significant clinical relevance, which added to the project's full integration into healthcare delivery. Telemedical consultation by specialized physicians is particularly important in rural areas, where specialized paediatric expertise is not always readily available [31–35]. An important result of the tele-paediatric network that the transfer to a more distant hospital could often be avoided. Most children could remain in the local clinic after the telemedicine consultation. This has several positive implications. First, it reduces stress and strain for families who would otherwise face the transfer of their child to an often-far-off specialized centre. Second, it alleviates the burden on tertiary paediatric hospitals due to unnecessary referrals, enabling them to focus on more complex cases. Third, it leads to a more efficient use of resources in regional clinics, as they are able to treat more patients with a broader range of conditions. Moreover, the high level of parental satisfaction with the telemedical services, as shown in a separate study, highlights the acceptability and perceived value of the intervention from the families’ perspective [16]. This reinforces the potential of telemedicine to support paediatric care delivery, especially in underserved rural regions. Economic analyses conducted as part of the project additionally revealed that the provided telemedicine services have significant impacts on the cost structure. Despite potential revenue losses for tertiary care providers, telemedical management of cases in hospitals of lower care levels could generate additional revenue, which in the long term could lead to cost savings from the statutory insurance and the societal perspective [not published yet]. The limited use of certain functionalities, such as telemedical triage, highlights the importance of organizational structure and role clarity within telemedicine networks. Our findings suggest that the optimal form of coordination may vary depending on the clinical context. For highly specialized consultations requiring specific expertise, a decentralized approach appears beneficial—particularly when clinicians already know each other and maintain established communication pathways. In such cases, direct collaboration can enhance responsiveness and trust. Conversely, in more general clinical scenarios, during emergency triage, or when the involved professionals are unfamiliar with one another, a centralized coordination structure may facilitate more systematic use and ensure effective allocation of telemedical resources. Future developments of the network might therefore consider a hybrid model, adjusting the level of coordination according to the degree of specialization and familiarity among care providers.

The complete participation of all paediatric clinics in the regions of Vorpommern-Greifswald and Vorpommern-Rügen suggests a structural interest and a perceived need for telemedical collaboration. This impression is further supported by accompanying qualitative and quantitative surveys on physician satisfaction and acceptance, the results of which will be presented in a separate analysis [36]. To the best of our knowledge, no studies have investigated the implementation and acceptance of comprehensive tele-paediatric care networks encompassing a broad spectrum of paediatric subspecialties aimed at supporting cross-organizational patient pathways in rural, underserved regions.

The implementation of a tele-paediatric network in a rural region in the northeast of Germany clearly demonstrates that telemedicine approaches can be a relevant option to supplement the care of children and adolescents. It reveals significant clinical and structural benefits, particularly regarding therapy optimization and the reduction of patient transfers. The integration of specialized expertise via telemedicine holds substantial potential to enhance the quality of care in rural areas while simultaneously reducing the burden on families and central healthcare institutions. The observed usage patterns highlight the importance of adaptable and flexible network models that accommodate both clinical and organizational needs. A hybrid coordination model—combining decentralized structures for highly specialized consultations with centralized triage and general support functions—could enhance both efficiency and usability. Sustained and expanded telemedical collaborations, particularly in subspecialties such as neuropaediatrics and paediatric surgery, should be prioritized. Future research should aim to optimize network coordination, clarify roles, and expand functionalities that address chronic care and patient-centered access. Controlled and prospective study designs would be one way to confirm these findings and facilitate broader scalability.

### Limitations

The recruitment of participating hospitals and patients was strongly dependent on the commitment of the treating physicians within the network as well as the individual hospital structures. These factors influence both the number of participating institutions and the frequency of telemedicine functionality usage. Therefore, the data on utilization and treatment occasions should be interpreted with due caution. Furthermore, recruitment rates varied significantly between different hospitals, leading to an unequal representation of specialties. Some subspecialties are underrepresented in the data, as they were integrated into the RTP network at a later stage.

A further limitation concerns the documentation of telemedicine contacts (cases) on the eHealth platform, which was not consistently complete. In particular, suspected diagnoses were missing in 35.1% of cases. This reflects the real-world conditions of the implementation phase, during which documentation practices varied and were not obligatory. Several structural and procedural factors likely contributed to this, including the asynchronous onboarding of hospitals and subspecialties, unequal implementation timelines, varying levels of familiarity with the eHealth platform, and different usage patterns based on clinical needs. These conditions may have led to an overrepresentation of certain diagnostic categories (e.g., neurological conditions, due to the early involvement of paediatric neurology) and an underrepresentation of others, introducing a structural selection bias. Consequently, the reported diagnostic spectrum should be interpreted with caution. Future studies using a prospective and controlled study design will be needed to systematically address such variability and minimize documentation bias.Additionally, the start of the project on April 1, 2020, coincided with the onset of the COVID-19 pandemic in Germany. The pandemic led to major disruptions in hospital operations, as many clinics were forced to prioritize internal processes, which significantly restricted research activities and affected data collection. The nationwide lockdown also resulted in a decrease in hospital admissions [37], thereby reducing the number of cases available for study inclusion. On the other hand, the heightened demand for telemedicine during the pandemic facilitated the adoption of digital solutions, potentially accelerating the integration of telemedicine into clinical workflows and lowering institutional barriers.

Due to the absence of a control group, causal relationships between the intervention and outcomes cannot be conclusively established. The findings should thus be interpreted as associations observed under real-world conditions.

## Conclusion

The implementation of a tele-paediatric network in the northeast of Germany demonstrated that telemedicine can significantly enhance healthcare provision for children and adolescents in rural areas, particularly by improving access to specialized care. While telemedical triage faced challenges, specialist video consultations proved to be highly effective, especially in the fields of paediatric neurology. The integration of telemedicine into routine care helped optimize treatment plans and reduce the need for transfers to distant hospitals, benefiting both patients and the healthcare system.

## Data Availability

Data is provided within the manuscript or supplementary information files.
